# Long‐term intermittent catheterization: Safety and outcomes from a prospective observational study

**DOI:** 10.1002/bco2.70200

**Published:** 2026-04-16

**Authors:** Peter Taffo, Gerard Amarenco, Carina Andersson, Nicola Morris, Jan Hörling

**Affiliations:** ^1^ Wellspect HealthCare Mölndal Sweden; ^2^ Service de Neuro‐Urologie et Explorations Périnéales Hôpital Pitié‐Salpêtrière Paris France; ^3^ Norrlands Universitetssjukhus Neurorehab, Neurocentrum Umeå Sweden; ^4^ Bristol Urological Institute (BUI), Southmead Hospital Bristol UK

**Keywords:** clinical study, intermittent catheterizationLoFriclong‐term safetyquality of lifeurological complications

## Abstract

**Objectives:**

The aim of this study was to evaluate long‐term safety, urological complication rates and quality of life (QoL) associated with urethral intermittent catheterization (IC) over a 5‐year follow‐up in adult, established, experienced subjects with longstanding IC use.

**Patients and Methods:**

Prospective, noninterventional, observational cohort study at three European sites. Adults (≥18 years) performing urethral IC with LoFric™ catheters for ≥6 years prior to enrolment were followed for 5 years. Outcomes included symptomatic urinary tract infections (UTIs), urethral strictures, bladder stones, prostatitis, epididymitis, patient‐reported outcomes (PROs) and perception of catheters and IC. QoL, including pain and discomfort, anxiousness and depression, self‐care and performing usual activities, was evaluated through the EQ 5DL questionnaire.

**Results:**

Ninety‐eight participants were enrolled (April 2015–August 2018); 49 completed 5‐year follow‐up (PP). Median age at inclusion was 61 years; 69% were male. Median follow‐up was 5 years; mean prior LoFric use was 13 years (range 6–30). Neurological conditions comprised 51% of underlying aetiologies. Across 5 years, there were no significant changes in UTI frequency, other urological complications, EQ‐5D tariff, or PRO ratings. At baseline, 59% (PP) reported ≥1 UTI in the prior 12 months versus 55% at Year 5 (*p* = 0.8036); mean UTIs decreased from 3.93 to 3.63 (*p* = 0.5434). Urethral stricture reports rose from 6% to 12% (NS). EQ‐5D tariff remained stable (mean 0.86 at inclusion and Year 5).

**Conclusions:**

In adults with more than a decade of prior IC experience, continued use of hydrophilic LoFric catheters over an additional 5 years was associated with unchanged complication rates and stable QoL and PROs, supporting the long‐term safety of IC as a bladder management strategy.

## 1 INTRODUCTION

Intermittent catheterization (IC) is widely recognized as the recommended first‐line strategy for bladder emptying in both neurogenic and non‐neurogenic lower urinary tract dysfunction. Compared with indwelling catheters, IC is associated with lower rates of urinary tract infections (UTIs), reduced risk of upper urinary tract deterioration and better long‐term renal preservation.^1^, ^2^ The introduction of hydrophilic single‐use catheters has further advanced IC safety by minimizing urethral microtrauma, reducing insertion friction and mitigating infection risks.^3^, ^4^, ^5^, ^6^, ^7^, ^8^, ^9^, ^10^, ^11^ Despite these improvements, bacteriuria and symptomatic UTIs continue to represent common and clinically relevant complications among IC users.^10^, ^12^

Beyond its clinical efficacy, IC has meaningful implications for patients' quality of life. Individuals performing IC frequently report greater satisfaction with bladder management compared with those relying on indwelling catheters, often citing enhanced autonomy, comfort and social participation, as well as less mechanical discomfort and inconvenience.^13^, ^14^ Such benefits make IC not only a medical intervention but also a critical contributor to overall functional independence and psychosocial well‐being.

While the short‐term safety and tolerability of hydrophilic IC are well established, robust prospective evidence on long‐term outcomes remains limited. Early prospective research by Wyndaele and Maes^15^ demonstrated favourable results over a mean follow‐up of 7 years, highlighting the feasibility of sustained IC use without significant renal compromise. Subsequent retrospective cohort studies spanning 5–9 years have supported these findings, showing low incidences of upper tract deterioration and acceptable rates of UTIs among users of hydrophilic catheters.^15^, ^16^ However, longitudinal data have also revealed variable burdens of bacteriuria and recurrent UTIs.^12^, ^17^ Reported adverse events of long‐term IC include urethral trauma, stricture formation and epididymitis,^3^, ^18^ underscoring the importance of continued long‐term evaluation to better define safety profiles and optimize management strategies.^19^

The present prospective study addresses this evidence gap by evaluating the 5‐year safety and patient‐reported outcomes (PROs) among adults who had already been performing IC—predominantly with hydrophilic LoFric catheters—for an average of more than a decade at baseline. By focusing on an experienced IC population, the study aims to characterize real‐world, long‐term use patterns and to provide a comprehensive assessment of both clinical safety and quality‐of‐life trajectories over time.

## 2 PATIENTS AND METHODS

### 2.1 Study design and population

This noninterventional, prospective, observational cohort study evaluated PROs and clinical outcomes in adults performing IC with LoFric intermittent urinary catheters. Clinical practice dictated catheter prescription and use. Three coordinating sites—France, Sweden and the United Kingdom—enrolled men and women aged ≥18 years who had used LoFric catheters for ≥6 years prior to study entry. Exclusion criteria were indwelling catheter use, recent urological surgery (<6 months) or active urological malignancy. Informed consent was obtained from each participant prior to the initiation of any study‐specific procedures.

Participants received a unique Subject ID and secure access to a study portal for electronic questionnaires. Assessments occurred at baseline and at approximately 1, 3 and 5 years (with 8‐, 4‐ and 4‐week windows, respectively). Demographics; urological complications (symptomatic UTIs, strictures, bladder stones, prostatitis and epididymitis); IC use patterns; perceptions; and adherence were collected via PROs. QoL was measured with EQ‐5D‐3L (questionnaire and EQ visual analogue scale [VAS]), a vertical numerical rating scale ranging from 0, indicating the worst imaginable health, to 100, indicating the best imaginable health.^20^

### 2.2 Outcomes

Primary outcomes were changes in self‐reported symptomatic UTI incidence and other urological complications over 5 years. Secondary outcomes included EQ‐5D tariff and domain‐level changes (mobility, self‐care, usual activities, pain/discomfort and anxiety/depression), as well as treatment satisfaction and adherence.

### 2.3 Statistical analysis

Analyses followed both ITT (all eligible enrollees) and PP (participants with baseline and 5‐year data) approaches. Descriptive statistics summarized baseline characteristics. Ordered categorical data (including 4−/5‐grade scales treated as continuous) used Wilcoxon signed‐rank tests; dichotomous data used McNemar's test. Proportions were tested with the binomial tests. Longitudinal renal function (where available) was analysed via repeated‐measures ANOVA. Significance was set at *p* < 0.05.

## 3 RESULTS

### 3.1 Participant disposition and baseline characteristics

Ninety‐eight adults were enrolled between April 2015 and August 2018 across three sites in France, Sweden and the United Kingdom (study duration extended to ~9.5 years due to slow recruitment). The PP set comprised 49 participants who completed the 5‐year evaluation. There were no statistically significant demographic differences or underlying reasons for catheterization at baseline between the ITT and the PP set. Mean age at inclusion was 61 years; 69% were male. Underlying aetiologies were primarily neurological (≈51%; including multiple sclerosis 19%, paraplegic spinal cord injury 16% and spina bifida 5%), followed by urological (22%) and surgical (9%) conditions; 14% reported other reasons. At inclusion, participants had performed IC for a mean of 14 years (range 6–34), using LoFric catheters for a mean of 13 years (range 6–30) (Table 1).

**TABLE 1:** Subject population and disposition.

		ITT (*n* = 98)		PP (*n* = 49)	
Demographic characteristics					
Sex (*n* and % of subjects)	Male	68	(69%)	34	(69%)
	Female	30	(31%)	15	(31%)
Age (years)	*N*	79		47	
	Missing	19		2	
	Mean (SD)	57	(13.09)	57	(12.36)
	Median	60		3	
	Range	18 to 79		18 to 79	
Main reason for catheterization					
Neurological		40	(54%)	25	(51%)
Urological without any underlying neurological condition or injury		16	(22%)	10	(20%)
Surgical without any underlying neurological condition or injury		7	(9%)	5	(10%)
Other		10	(14%)	8	(16%)
Do not know		1	(1%)	1	(2%)
Missing		20	(NA)	0	(NA)
Years of LoFric use for IC					
*N*		74		49	
Missing		24		0	
Min		6		6	
Median		12.50		13.00	
Max		30		30	
Mean (SD)		12.97	(4.87)	13.43	(5.27)

No statistical difference in the EQ‐5D‐3L scores or urological complications were observed at baseline between the ITT and PP set (Table 2).

**TABLE 2:** Objectives and outcome variables.

Objective and outcome	Baseline ITT, 98 subjects	Baseline PP, 98 subjects	Follow‐up 3, PP 49 subjects
UTI last 12 months	61%	59%	55%
#UTIs per year	Mean 3.51; range 1–12	Mean 3.93; range 1–12	Mean 3.63; range 1–12
Antibiotics due to UTI	Mean 3.21; range 1–10	Mean 3.44; range 1–10	Mean 3.75 range 1–16
Hospitalization due to UTI	9%	7%	7%
Urosepsis	3%	2%	0%
Urethral Strictures	7%	6%	12%
Prostatitis	8% of males	9% of males	3% of males
Epididymitis	4%	3%	6%
Bladder stones	1%	1%	4%
Other urol. Complications	7%	8%	10%
EQ VAS mean +/−STD	70.24+/−20.43	73.60+/−19.27	71.90+/−21.21
Pain/discomfort	42% No pain 42% Some pain 15% Extreme pain	41% No pain 47% Some pain 12% Extreme pain	38% No pain 56% Some pain 6% Extreme pain
Anxiety/depression	59% No anxiety 38% Moderate anxiety 3% Extreme anxiety	57% No anxiety 39% Moderate anxiety 4% Extreme anxiety	63% No anxiety 33% Moderate anxiety 4% Extreme anxiety
Self‐care% no problem.	82%	88%	88%
Usual activities; no problem	65%	71%	77%
Mobility; no problems	58%	63%	56%
QoL summary tariff mean +/−STD	0.85 +/−0.14	Mean 0.86 +/−0.13	Mean 0.85 +/−0.12
Satisfaction with catheter, agree or strongly agree		94%	100%

Of the 74 subjects who reported a medical background at baseline, 35% had previously received Botulinum toxin A (Botox®) injections for bladder management, 19% routinely used prophylactic (preventive) antibiotics, and 20% had undergone bladder surgery. Five subjects (7%) reported having a reconstructed bladder.

At the 5‐year follow‐up visit, no statistically significant differences were observed between the baseline population and the per‐protocol (PP) set. However, descriptively, two additional subjects were receiving prophylactic antibiotics, three subjects with a history of bladder surgery were lost to follow‐up, and one additional subject reported a reconstructed bladder. There was also a slight trend toward decreased normal hand function, increased urethral sensitivity and a higher number of catheters used per day. A summary of these findings is provided in Table 3.

**TABLE 3:** Medical background at baseline and follow‐up.

		Baseline *n* (% of 74)	5‐year FU *n* (% of 49)	Comment
Botox injections	ITT	26 (35%)	16 (33%)	No additional Botox injections
	PP	16 (33%)		
Prophylactic antibiotics	ITT	14 (19%)	12 (24%)	Two more subjects on prophylactic antibiotics
	PP	10 (20%)		
Bladder surgery	ITT	15 (20%)	8 (16%)	Three subjects with bladder surgery lost to follow‐up
	PP	11 (22%)		
Bladder reconstruction	ITT	5 (7%)	3 (N/A)	One subject having bladder reconstruction during the period
	PP	2 (N/A)		
Normal hand function	ITT	61 (82%)	32 (65%)	Nonsignificant decrease in hand function
	PP	41 (84%)		
Normal urethral sensitivity	ITT	33 (45%)	26 (53%)	Nonsignificant increase in urethral sensitivity
	PP	22 (45%)		
Number of catheterizations per day	ITT	5.18	5.45	Nonsignificant increase of catheterizations per day
	PP	5.08		

### 3.2 Urinary tract infections

In the PP set, 59% of participants reported at least one symptomatic UTI in the 12 months preceding baseline, compared with 55% at Year 5 (*p* = 0.8036). Female participants had a higher incidence of UTI than males: at baseline, 67% of women and 56% of men reported at least one UTI, while at Year 5, the proportion remained 67% among women and decreased slightly, though not significantly, to 50% among men.

When stratified by indication, 60% of participants with a neurological condition and 58% of those with a non‐neurological urinary condition reported a UTI at baseline. At the 5‐year visit, the corresponding proportions were 48% (not significant) in the neurological group and 62% in the non‐neurological group.

Mean UTIs over 12 months were 3.93 at baseline and 3.63 at Year 5 (*p* = 0.5434). At baseline, 97% received treatment for UTIs versus 93% at Year 5 (*p* = 1.000). Antibiotics were the most common therapy, with mean courses of 3.44 at baseline and 3.75 at Year 5. Urosepsis within 12 months pre‐baseline was reported by 3% (ITT). Hospitalization for UTI occurred in 7% in the 12 months prior to baseline and 7% at Year 5.

The mean number of catheterizations per day did not change significantly over the period being 5.08, 5.73, 5.29 and 5.45 at baseline, visit 1, 2 and 3, respectively, ranging from 1 to 10 per day.

### 3.3 Other urological complications

Reports of urethral stricture increased from 6% at baseline to 12% at Year 5 (NS; *p* = 0.3750). Among male participants, prostatitis and epididymitis were reported by 8% and 4% at baseline, respectively, and 3% and 6% at Year 5 (both NS). Bladder stones were reported by 1% at baseline and 4% at Year 5 (NS). Other complications (7% at both time points) included urethral calcification, bleeding during IC, significant kidney infections, benign prostatic hyperplasia and urine leakage related to a spinal column cyst.

### 3.4 Treatment satisfaction and quality of life

EQ‐5D tariff remained stable. In the ITT set, mean tariff at inclusion was 0.85 (range 0.435–1.035); in the PP set, 0.86 (range 0.597–1.000) at inclusion and unchanged at Year 5. Pain/discomfort shifted modestly from ‘extreme’ to ‘some’ without statistical significance: At inclusion, 42% reported no pain/discomfort, 42% some and 15% extreme; at Year 5, 38%, 56% and 6%, respectively. Anxiety/depression ratings were similar over time (not anxious/depressed 57% to 63%; moderate 39% to 33%; extreme 4% to 4%). Self‐care and usual activities did not change significantly (Table 2).

Catheter satisfaction was high and stable. At baseline, 94% agreed/strongly agreed that they were very satisfied with their catheter; at follow‐up, 100% agreed/strongly agreed. Other satisfaction items remained between 80% and 100% agree/strongly agree throughout. No significant difference between the ITT and the PP set. Data summarized in Table 4.

**TABLE 4:** User satisfaction with catheterization.

	PP = 49	Completely agree	Agree	Do not agree	Absolutely not agree
I am satisfied with my catheter	Baseline	61%	33%	6%	0%
	5‐year FU	67%	33%	0%	0%
It is easy to prepare the catheter for each use	Baseline	67%	31%	2%	0%
	5‐year FU	71%	24%	4%	0%
I feel confident in my ability to use my catheter	Baseline	80%	20%	0%	0%
	5‐year FU	78%	22%	0%	0%
Carrying my daily need of catheter is manageable	Baseline	45%	41%	14%	0%
	5‐year FU	61%	33%	6%	0%
I can catheterize in a discrete way when away from home	Baseline	35%	49%	8%	8%
	5‐year FU	47%	41%	12%	0%
Catheter makes me feel safe when away from home	Baseline	47%	43%	4%	6%
	5‐year FU	53%	43%	4%	0%
I feel confident that my catheter always empties my bladder completely	Baseline	59%	37%	4%	0%
	5‐year FU	61%	37%	2%	0%
My catheter helps me function well in my daily life	Baseline	49%	45%	6%	0%
	5‐year FU	61%	35%	4%	0%

## 4 DISCUSSION

One of the major concerns for people practicing IC is the safety of the procedure over time. In a study by Hao et al.,^21^ 75.5% of the 327 responders in a survey indicated that they strongly agreed or agreed to the statement that over time catheterization will damage my urethra; hence, data confirming long‐term safety of IC are warranted.

The findings of this long‐term, noninterventional study provide important real‐world evidence supporting clinical safety, tolerability and sustained patient satisfaction associated with hydrophilic catheters for intermittent catheterization (IC). Participants—who had already been performing IC with LoFric hydrophilic catheters for an average of 13 years at baseline—reported no significant changes in the type or frequency of urological complications, perceived quality of life or attitudes toward IC over the 5‐year follow‐up period, despite the expected effects of aging within the cohort. These results reinforce prior long‐term observations demonstrating stable infection profiles, minimal upper urinary tract complications and durable usability with hydrophilic low‐friction catheter technology.^12^, ^15^, ^16^, ^17^, ^18^ Of note is that UTIs were patient‐reported and not microbiologically confirmed, reflecting perceived and treated infections rather than strictly defined bacteriuria.

The prevalence of participants reporting at least one UTI in the preceding year did not increase over time and mean annual UTI counts remained stable. At baseline, 61% of subjects reported at least one UTI in the prior 12 months, compared with 55% at follow‐up. These values are consistent with previously published long‐term data, where approximately half of chronic IC users experienced at least one UTI within 6–12 months (e.g., 52%^19^ and 54%^22^). This stability in infection frequency over an additional 5 years of follow‐up suggests that hydrophilic catheter use may mitigate cumulative risk despite prolonged duration of IC practice.

The modest, nonsignificant increase in reported urethral strictures (from 6% to 12%) aligns with evidence that stricture risk can accumulate gradually with advancing age and cumulative catheterization frequency.^18^, ^23^ Notably, the incidence observed in this study remained comparatively low, even among participants with a mean prior IC duration of 13 years at study entry. In contrast, higher stricture rates have been reported in predominantly neurogenic populations followed over longer periods,^23^ suggesting that patient characteristics and technique may modulate long‐term risk. Rates of prostatitis and epididymitis were low and stable over time; the small decrease in prostatitis observed parallels the unchanged or slightly reduced UTI reporting, consistent with the known association between recurrent UTI and prostatic inflammation in men.^24^ Reported bladder stones were infrequent and did not differ significantly from baseline, further supporting the long‐term urological safety of hydrophilic IC.

At 5 years, the mean EQ‐5D‐3L index value (0.85) was identical to the ITT baseline value and only 0.01 lower than the PP baseline value. For the EQ VAS, the mean change from baseline was −0.7. Although no universally accepted minimally important difference (MID) exists for EQ‐5D‐3L, previous research has suggested that a deterioration of ≥−0.02 for the EQ‐5D‐3L index and ≥−6.5 for the EQ VAS may represent a meaningful decline in health status.^25^ In this study, no statistically significant differences were observed between baseline and follow‐up scores. These PROs indicated stable quality of life during the follow‐up period, consistent with prior evidence emphasizing the independence, comfort and sense of control that IC provides.^7^

The EQ‐5D‐3L index value of 0.85 closely mirrored previously published data (0.82) for IC users^22^ and remained higher than reported values for individuals using indwelling or suprapubic catheters, reinforcing the established preference for IC among patients requiring long‐term bladder management.

Satisfaction with LoFric hydrophilic catheters remained consistently high throughout the study, with all participants at the final follow‐up expressing agreement (33%) or strong agreement (67%) with the statement that they were satisfied with their catheter. These findings underscore the sustained acceptability and usability of hydrophilic‐coated catheters over extended use.

Taken together, these results add to the growing body of longitudinal evidence demonstrating that IC is a safe and effective long‐term method for bladder management. It supports continence, preserves upper urinary tract function and maintains patient quality of life over time. In counselling, this supports a clear message that for most patients, day‐to‐day life, functioning and overall health status can remain stable over years on IC, with many experiencing added benefits such as greater independence, comfort and control.

Overall, the findings support continued use of hydrophilic‐coated catheters as a preferred option to minimize infection risk, reduce urethral trauma and promote sustained user satisfaction in chronic IC populations.

## 5 LIMITATIONS

This study has several limitations that should be considered when interpreting the findings. The study design was noninterventional and relied solely on self‐reported data collected through questionnaires, which may introduce reporting bias. The absence of on‐site clinical assessments limits objective validation of events such as UTI, stricture or stone formation.

As only participants who remained alive and retained in follow‐up were eligible for these evaluations, the analytic population could increasingly reflect individuals with more favourable baseline characteristics, slower disease progression or greater therapy tolerance, and hence, the survivor cohort might not be fully representative of the original study population. However, in this study the EQ‐5D‐3L scores at baseline were not different between the subjects completing the studies and those lost at follow‐up.

Information about concomitant medication that might contribute to outcome, for example, anticholinergics and/or Botox, was not collected in this NIS.

Recruitment and retention were challenged during the COVID‐19 pandemic, yielding a smaller PP set and potentially reduced power and generalizability. Missing data could not be retrospectively recovered in this decentralized design.

Despite these limitations, the study provides valuable long‐term insights into PROs and satisfaction with LoFric catheter.

## 6 CONCLUSION

Long‐term use of hydrophilic LoFric catheters for IC was associated with low and stable rates of urological complications and with sustained QoL and satisfaction over 5 years in adults who had already performed IC for more than a decade. These results support the long‐term safety and usability of IC in real‐world practice.

## AUTHOR CONTRIBUTIONS

Peter Taffo is the project leader, Jan Hörling analysed the data and wrote the manuscript. All investigators had full access to data and have approved. Gerard Amarenco, Carina Andersson, Nicola Morris clinical investigators performing the study.

## CONFLICT OF INTEREST STATEMENT

PT and JH are employees of Wellspect Healthcare which sponsored this study. GA, CA and NM declare no conflicts of interest.
